# Non-Nutrient, Naturally Occurring Phenolic Compounds with Antioxidant Activity for the Prevention and Treatment of Periodontal Diseases

**DOI:** 10.3390/antiox4030447

**Published:** 2015-06-24

**Authors:** Alfonso Varela-López, Pedro Bullón, Francesca Giampieri, José L. Quiles

**Affiliations:** 1 Department of Physiology, Institute of Nutrition and Food Technology “José Mataix”, Biomedical Research Center (CIBM), University of Granada, Armilla, Avda. del Conocimiento s.n., 18100 Armilla, Spain; E-Mail: avarelalopez@gmail.com; 2 Department of Periodontology, Dental School, University of Sevilla, C/Avicena s.n., 41009 Sevilla, Spain; E-Mail: pbullon@us.es; 3 Department of Clinical Sciences, Marche Polytechnic University, Ancona 60100, Italy; E-Mail: f.giampieri@univpm.it

**Keywords:** anti-inflammatories, phytochemical, periodontitis, polyphenols

## Abstract

One of the main factors able to explain the pathophysiological mechanism of inflammatory conditions that occur in periodontal disease is oxidative stress. Given the emerging understanding of this relationship, host-modulatory therapies using antioxidants could be interesting to prevent or slow the breakdown of soft and hard periodontal tissues. In this context, non-nutrient phenolic compounds of various foods and plants have received considerable attention in the last decade. Here, studies focusing on the relationship between different compounds of this type with periodontal disease have been collected. Among them, thymoquinone, coenzyme Q (CoQ), mangiferin, resveratrol, verbascoside and some flavonoids have shown to prevent or ameliorate periodontal tissues damage in animal models. However evidence regarding this effect in humans is poor and only limited to topical treatments with CoQ and catechins. Along with animal experiments, *in vitro* studies indicate that possible mechanisms by which these compounds might exert their protective effects include antioxidative properties, oxygen and nitrogen scavenging abilities, and also inhibitory effects on cell signaling cascades related to inflammatory processes which have an effect on RNS or ROS production as well as on antioxidant defense systems.

## 1. Introduction

Periodontal diseases are chronic inflammatory conditions characterized by loss of connective tissue, alveolar bone resorption and formation of periodontal pockets as a result of the complex interaction between pathogenic bacteria and the host’s immune response [1]. At present, periodontitis, its destructive phase, is considered to be initiated and perpetuated by a small group of predominantly Gram-negative, anaerobic, or micro-aerophilic bacteria that colonize the subgingival area. Bacteria cause the observed tissue destruction directly by toxic products and indirectly by activating host defense systems, *i.e.*, inflammation [2]. Inflammation is a localized protective response of the organism to a noxious stimulus, whether mechanical, chemical, or infectious, which serves to destroy, dilute, or wall off both the injurious agent and the injured tissue [3]. Whether acute or chronic, inflammation is dependent upon regulated humoral and cellular responses, and the molecules considered to mediate inflammation at one time or another are legion [4]. As a consequence of the multitude of processes that it implies, inflammation entails the production of an amount of energy sufficient to support all necessary biochemical reactions and the output of all mediators, such as proteins. Most of the energy is produced in the mitochondria via the electron-transport chain that implies an oxidation process with oxygen as the main combustive agent [3].

One of the main factors able to explain the pathophysiological mechanism of inflammatory conditions that occur in periodontal diseases is oxidative stress [5], and, recently, its complex role in relation to periodontal breakdown has been comprehensively reviewed [6,7]. Reactive oxygen species (ROS) are essential to many physiological processes but when an antioxidant system is unable to efficiently counteract their action, tissue and cell damage can result, enhancing periodontal destruction [6]. This aspect is in accordance with a recent study reporting new evidence that chronic periodontitis, as a common potential source of low-grade inflammation, could be associated with a systemic oxidative stress state and reduced overall antioxidant capacity [8]. In this sense, *Porphyromonas gingivalis* lipopolysaccharide (LPS) was found to be responsible for high mitochondrial ROS and for mitochondrial dysfunction because it affected the amount of respiratory chain complex I and III [9,10]. Furthermore, high levels of mitochondrial-derived ROS, accompanied by mitochondrial dysfunction, in peripheral blood mononuclear cells (PBMCs) from patients with periodontitis [11] have been reported, and several studies have demonstrated an increase in products from oxidative damage in plasma and serum of subjects with periodontitis compared with healthy individuals [12,13,14]. Likewise, there is increasing evidence for a decreased antioxidant capacity in periodontal tissues and fluids of subjects with periodontitis [7,15,16]. Additionally, damage mediated by ROS in periodontal diseases also results from the physiological activity of phagocytosing leukocytes, *i.e.*, polymorphonuclear neutrophils (PMN) during the “respiratory burst” [6,17]. An event characteristic of mammalian inflammation, tissue infiltration by PMNs and monocytes and subsequent phagocytosis, features a burst of non-mitochondrial O_2_ consumption, which may be 10 or 20 times that of resting consumption. Oxygen uptake in neutrophils and macrophages is due to the action of a plasma-membrane-bound flavoprotein cytochrome b245 NADPH oxidase system that increases NADPH production via the hexose monophosphate shunt and generates superoxide anion radicals, hydrogen peroxide, hydroxyl radicals and hypochlorous acid, all capable of damaging either cell membranes or associated biomolecules [6]. Moreover, among the inflammatory mediators produced there is nitric oxide (NO) [18], which also is a highly reactive free radical. Due to this feature it can react with certain biological molecules or other reactive species leading to different cytotoxic nitrogenated compounds [19]. The interactions of NO with other oxidants are especially important since they may give rise to a free radical cascade and formation of reactive nitrogen species (RNS) such as peroxynitrite [20]. Consequently, a variety of molecular oxidative species appears in the inflamed tissues, including RNS and ROS. Actually, several forms of periodontal disease have been often associated with functionally activated PMN exhibiting increased ROS production [6]. Thus, the up-regulation of pro-inflammatory transcription factor in inflamed periodontal tissues contributes to antioxidant depletion and reactive molecular species generation [17,21]. Moreover, some oxidation products can increase neutrophil adhesion, chemotaxis and priming in hyper-reactive neutrophils, and might augment the damaging effects of the resultant oxidative stress [22,23,24].

Given the emerging understanding of the role of oxidative stress in periodontal disease, host-modulatory therapies using antioxidants could be interesting to prevent or slow the breakdown of soft and hard periodontal tissues [25]. Antioxidants are substances that, when present at low concentrations compared with oxidizable substrates, can significantly delay or prevent oxidation of those susceptible substrates [6]. Thus they constitute an efficient defense system against the oxidative damage induced by reactive molecular species [2,6]. Their specific role is to remove or inactivate reactive molecular species, or to repair damage caused by them. However, when the former fails to efficiently counteract reactive molecule action, tissue damage can result [6,17]. Based on the way they act [3], antioxidants are functionally classified as preventive antioxidants, radical scavengers and repair and *de novo* enzymes [3,6]. Natural products can play an important role in the prevention and treatment of periodontal diseases [26,27] because many of them have anti-oxidative and anti-inflammatory properties [25]. In this context, non-nutrient phenolic compounds of various foods and plants have received considerable attention in the last decade [28]. In order to explore this issue, a search without restrictions was conducted in the PubMed database (Nationtal Insitute of Health, Washington, DC, USA) combining the terms “antioxidant”, “periodontal disease” and “food”, and accessible items that evaluated only naturally occurring compounds both *in vivo* and *in vitro* were considered. After read them, it was found that possible mechanisms by which these compounds might exert their protective effects included antioxidant properties, oxygen and nitrogen scavenging abilities, but also inhibitory effects on cell signaling cascades related to inflammatory processes which have effect on RNS or ROS production as well as on antioxidant defense systems. As a result, it is not easy to differentiate between antioxidant and immunomodulatory effects. For this reason, in the present review, we collect different phenolic compounds whose possible role based on any of these mechanisms have been evaluated in relation to periodontal disease (Table 1).

## 2. Thymoquinone

Thymoquinone (2-isopropyl-5-methyl-1,4-benzoquinone), a benzoquinone derivative, is the main constituent of the volatile oil from *Nigella sativa* seeds. This compound has a range of pharmacologic properties, among these its antioxidant effect is considered to be one of its most significant. It has been reported that thymoquinone induces an antioxidant response through the scavenging capability of various free radicals, with its scavenging power being as effective against superoxide anions as superoxide dismutase [29]. Thymoquinone has also been argued to have anti-inflammatory potential through membrane lipid peroxidation, via eicosanoids [30]. In rats, ligature placement on mandibular first molars was associated with higher alveolar bone loss, an increase of inflammatory cell infiltrate and osteoclast number and a decrease of osteoblastic activity, which did not occur when animals simultaneously received thymoquinone by gastric feeding [25]. Considering both, anti-inflammatory and antioxidant properties, as well as the effects on alveolar bone loss and expression of proinflammatory cytokines, thymoquinone likely presents a new aspect in the prevention and treatment of periodontal disease preventing the initiation and progression of periodontitis.

## 3. Coenzyme Q

Coenzyme Q (CoQ) is a naturally occurring coenzyme formed from the conjugation of a benzoquinone ring with a hydrophobic isoprenoid chain of varying chain length, depending on the species [31]. In particular, the chemical nomenclature of CoQ_10_ is 2,3-dimethoxy-5-methyl-6-decaprenyl-1,4-benzoquinone that is in the trans configuration (natural) [32]. Due to its ubiquitous presence in nature and its quinone structure (similar to that of vitamin K), CoQ is also known as ubiquinone [33]. It is found in every plant and animal cell and is located in the inner membrane system of the mitochondria, other membranes and in plasma lipoproteins [34]. It exists in two molecular forms, ubiquinone, the oxidized form, and ubiquinol, the reduced form, which are the basis for its antioxidant properties [35]. Ubiquinone molecules are classified based on the length (n) of their isoprenoid side chain (CoQ_n_) [31]. CoQ_9_ is the predominant form in relatively short-lived species such as rats and mice whereas in humans and other long-living mammals the major homolog is CoQ_10_ [36].

Ubiquinone is also referred to as “coenzyme” because of its unique ability to participate in chemical reactions but remain at steady-state levels in the cell [37]. The well-recognized function of CoQ is mitochondrial energy coupling. It is an essential part of the cellular machinery used to produce ATP in the inner membrane of mitochondria where this process mainly takes place [34]. The other important function is that it acts as a primary scavenger of free radicals as it is well located in the membranes in close proximity to the unsaturated lipid chains. Less well-established functions also include oxidation/reduction control of signal origin and transmission in cells, which induce gene expression, control of membrane channels, membrane stabilization and lipid solubility [34]. As it is an antioxidant, CoQ has received much research attention in the medical literature in the last several years [38]. Studies have demonstrated that CoQ has anti-oxidative effects and anti-aging properties at the skin level [39]. Among its possible applications, CoQ is recommended as a supplement to traditional therapy for cardiovascular diseases [40]. 

Human cells are able to synthesize CoQ_10_ from the amino acid tyrosine, in an eight-step aromatic pathway, requiring adequate levels of vitamins such as folic acid, niacin, riboflavin and pyridoxine [41]. Since CoQ_10_ is synthesized *de novo* in all tissues, it is presumed that under normal circumstances they are not dependent on an exogenous supply of CoQ_10_. However, situations may arise in which the body’s synthetic capacity is insufficient to meet CoQ_10_ requirements. Susceptibility to CoQ_10_ deficiency appears to be greatest in cells that are metabolically active (such as those in the heart, immune system, gingiva and gastric mucosa), since these cells presumably have the highest requirements for CoQ_10_ [33]. A deficiency of CoQ_10_ has been found in the gingival tissue from patients with periodontal disease [42,43], as well as in their peripheral blood mononuclear cells (PBMCs) [11]. Nevertheless, CoQ_10_ deficiency in the gingiva may exist independently of and/or because of periodontal disease [42,44]. Moreover, it has also been found that CoQ_10_ remained unchanged in the periodontally affected tissues during gingivitis despite other antioxidant, such as vitamin E, being dramatically decreased [45]. If a deficiency of CoQ_10_ existed in gingival tissue for nutritional causes and independent of periodontal disease, then the advent of periodontal disease could enhance the gingival deficiency of CoQ_10_ [42]. In such patients, oral dental treatment and oral hygiene procedures can remove just the local factors (plaque and calculus) but cannot correct the systemic deficiency of CoQ_10_ [42]. Thus, it has been suggested that mechanical periodontal therapy along with the adjunctive use of CoQ should be included for an improved treatment of this type of periodontal disease [42,44].

Many clinical trials with oral administration of CoQ to patients with periodontal disease have been conducted. The results have shown that oral administration of CoQ_10_ increases the concentration of CoQ_10_ in the diseased gum and effectively suppresses periodontal inflammation [46,47]. Furthermore, they indicated that CoQ_10_ might reduce gingival inflammation without affecting total antioxidant levels in the gingivocrevicular fluid [48]. Oral administration of CoQ combined with vitamin E has also shown a beneficial effect on the periodontal tissue, since periodontal pockets shallowed by 30% accompanied by a decrease of several clinical outcomes (including plaque index, interdental hygiene index, gingival index and sulcus bleeding index) [49]. In addition to oral administration, other uses of CoQ also offer promising results. In rats, ubiquinol (reduced form of CoQ) applied topically on the gingival surface suppresses age-related inflammatory reactions, the mechanism for this is suggested to be the lower gene expression of caspase-1, interleukin(IL)-1β and Nod-like receptor protein 3 inflammasomes in the periodontal tissue. It also leads to a differentiation of osteoclasts associated with aging, probably by inhibiting oxidative stress as indicated by a decreased oxidative DNA damage [50]. Topical application is a convenient method in the dental clinical setting, and clinical trials evaluating benefits of CoQ topical (*i.e.*, extrasulcular) application (in gel-form) also exist in humans [38,51,52]. Moreover, intra-pocket application was tested as well in the same trials [38,51]. These studies showed that both types of application of a gel containing CoQ_10_ for 6 weeks improved major clinical parameters. Further, CoQ_10_ use has also been tested after a scaling and root planning treatment [38,51], and its application combined with mechanical treatment offered better results, but only when the gel was applied to the periodontal pockets [38].

## 4. Gallic Acid

Gallic acid (3,4,5-trihydroxybenzoic acid) is an endogenous plant phenolic acid found abundantly in tea, grapes, berries and other fruits as well as in wine [53,54,55]. It is also found in some hard wood plant species such as oak (*Quercus robur*) or chestnut (*Castanea sativa* L.) [56,57]. Among others, gallic acid is known to have strong antioxidant [58] and anti-inflammatory [59] properties. Treatment with this compound in non-toxic concentrations revealed a strong inhibitory effect on NO production without affecting iNOS expression in murine macrophage-like cells (RAW 264.7 cells) stimulated with LPS of *Aggregatibacter actinomycetemcomitans* (previously *Actinobacillus actinomycetemcomitans*) or *Fusobacterium nucleatum*, two major bacteria implicated in periodontitis [60]. However, it slightly increased ROS production in them. In spite of these effects, it did not show stronger activity than other phenolic compounds also tested in the same assay, more research on gallic acid therapeutic use could lead to interesting results due to its simplicity and availability.

## 5. Hydroxytyrosol

Hydroxytyrosol (3,4-dihydroxyphenylethanol) is a phenylethanoid [61] that comes from the hydrolysis of oleuropein, which originates during the maturation of olives, storage of the oil and preparation of table olives. These processes give rise to hydroxytyrosol along with oleuropein aglycone and elenolic acid, that are responsible, in part, for the complex and varied flavor of the oil and olive [62]. As a consequence of their polar character, phenolic compounds such as hydroxytyrosol are found in great quantities in the remains from oil processing, such as pomace olive oil, olive-mill waste water, or the rinse waters. For this reason, byproducts from olive oil production constitute a major source of hydroxytyrosol. Nevertheless, hydroxytyrosol also has an amphipathic behavior, and the molecule is therefore found in olive oil [62,63]. Additionally, it is also located in the olive leaf, where it is accompanied by other phenolic compounds such as oleuropein [64]. To date, hydroxytyrosol has been demonstrated in numerous studies to have antiatherogenic, anti-inflammatory and antitumor effects, which have been widely reviewed [65]. Regarding the periodontum, a study in rabbits reported a certain anti-inflammatory effect of supplementation with hydroxytyrosol. Specifically, it was reported that it reduced endothelial activation in rabbits that had developed atherosclerosis induced by a diet rich in saturated fats, but it did not improve endothelial activation and lower cellularity in gingival mucosa, which are also associated with this pathology [66]. However, there is no data regarding its effect on alveolar bone or periodontal oxidative stress levels. In that sense, a more recent study has shown that a virgin olive oil-based diet decreased alveolar bone breakdown associated with aging in rats, which is associated with lower gingival lipid peroxidation and increased systemic levels of major proinflammatory cytokines [67]. At present, it is well established that the health benefits of olive oil are not concentrated solely in its fatty-acid content [65], since it has been noted that refined olive oil (free of phenols) lacks the antioxidant effects present in virgin olive oil [68,69], whereas the phenolic fraction of olive oil considerably improves the lipid peroxidation of LDL lipoproteins, which is related to an increase in antioxidant capacity [70].

The oil also includes several bioactive compounds with high antioxidant capacity, such as hydroxytyrosol [71,72].

## 6. Mangiferin

Mangiferin is a glycosylated xanthone (C2-b-d-glucopyranosyl-1,3,6,7-tetrahydroxyxanthone), generally called C-glucosyl xanthone, widely distributed in higher plants [73,74], and it is the predominant constituent (10%) of mango (*Mangifera indica* L.) extract which belongs to the family Anacardiaceae [75,76]. This plant, which grows in tropical and subtropical regions, is widely used in folk medicine for various therapeutic indications [75]. Based on the various studies done on mangiferin, it possesses a wide range of pharmacological actions including anti-inflammatory [77], antioxidant [76], antidiabetic [78], immunomodulatory and antitumor effects [79]. Based on this bioactivity exhibited by mangiferin, growing attention has been given to its medical significance for human health [80]. In particular, it has been demonstrated that it has bone anti-resorption activity in lumbar vertebrae in an experimental model using a dose of 100 mg/(kg·day) [81]. Oral administration of mangiferin significantly reduced alveolar bone loss in a rat model after periodontitis induction by ligatures placement. This could be due to its inmunomodulatoy effects since bone loss prevention was accompanied by a reduction of cellularity, a decrease in COX-2 expression and an inhibition of rolling and adhesion of leukocytes. Curiously, mangiferin-treated rats presented an earlier peak of cell proliferation and augmented angiogenesis in the injured region [82] than controled animals.

## 7. Resveratrol

Resveratrol (*trans*-3,4ʹ,-5-trihydroxystilbene) is a widely available stilbenoid found in several plants, including berries, peanuts [83,84,85] and, in particularly high concentrations, grape skins and red wine [86]. Numerous studies on resveratrol have demonstrated a variety of biologic properties with health benefits [87,88,89,90]. Among these, it has also been shown that resveratrol may positively interfere with osteoblastogenesis, contributing to new bone formation [91]. These properties could be very useful for periodontitis treatment. Resveratrol administration in drinking water has shown to lead to relieved alveolar bone resorption associated with ligature placement [92]. Further, continuous systemic administration of resveratrol also decreased periodontal breakdown induced by similar methods [28], confirming the positive effects of resveratrol on these types of models.

Traditionally, many of the health benefits of resveratrol have been attributed to its antioxidant properties, as it is a known scavenger of superoxides, hydroxyl radicals and peroxynitrites [84]. In addition, resveratrol induces antioxidant enzyme activities [87,93] and activates the nuclear factor E2-related factor (Nrf2) antioxidant defense pathway. When Nrf2 is activated, it translocates to the nucleus, where it mediate the transcription of target genes which enhances cellular resistance to oxidative stress and confers protection against inflammation such as heme oxygenase 1 (HO-1) and NAD(P)H:quinine oxidoreductase 1 (NQO-1) [94]. In that sense, resveratrol also enhances mitochondrial biogenesis through the activation of AMP-activated protein kinase (AMPK) via sirtuin 1 (Sirt1), an NAD-dependent nuclear class III histone deacetylase [95,96]. The combination of these mechanisms would reduce ROS levels and mitochondrial dysfunction explaining that in the periodontitis rat models cited, alveolar bone loss prevention by resveratrol was associated with the improvement of the systemic levels of different oxidative stress markers including 8-hydroxydeoxyguanosine (8OHdG), dityrosine and nitrotyrosine [92]. In addition, down-regulation of the inducible NO synthase (iNOS) expression by resveratrol was also noted [97] reducing NO production, which would collaborate in oxidative stress reduction. This last point could result from resveratrol’s immunomodulatory effect since the systemic levels of some proinflammatory cytokines also decreased [28,92]. Therefore, according to animal experiments results, resveratrol might prevent the progression of periodontal disease, presenting a promising, innovative host-modulatory therapeutic approach in the treatment of periodontitis [28].

## 8. Verbascoside

**Figure 1:**
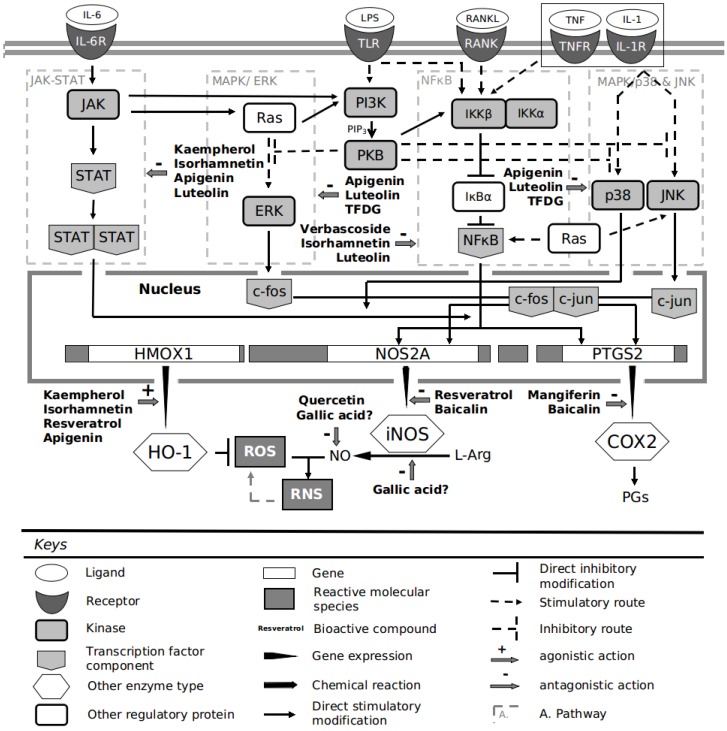
Main phenolic compound effects on cell signaling pathways and enzyme implicated on oxidative/nitrosative stress and inflammation. *Acronyms and Abbreviations*. COX2: Cyclooxygenase, ERK: Extracellular signal-regulated kinases, HO-1: Heme oxygenase 1, IKKα: IB kinaseα, IKKβ: IκB kinase β, IL-1: Interleukin 1, IL-1R: Interleukin 1 receptor, IL-6: Interleukin 6, IL-6R: Interleukin 6 receptor, IκBα: Inhibitor of κ light polypeptide gene enhancer in B-cells α, JAK: Janus kinase, JNK: c-jun terminal kinase, l-Arg: l-Arginine, MAPK: Mitogen-activated protein kinases, NfκB: Nuclear factor kappa-light-chain-enhancer of activated B cells, LPS: Lypopolysaccharide, PGs: Prostaglandins PI3K: Phosphoinositide 3-kinase, PKB: Protein kinase B, RANK: Receptor activator of nuclear factor κB, RANKL: Receptor activator of nuclear factor κB ligand, RNS: Reactive nitrogen species, ROS: Reactive oxygen species, STAT: Signal transducer and activator of transcription, TFDG: Theaflavin digallate, TLR: Toll-like receptor 2/4, TNF: Tumor necrosis factor α, TNFR: Tumor necrosis factor α receptor.

Verbascoside or acetoside is a phenylpropanoid glycoside isolated from some *Lamiaceae* plants, which is structurally characterized by a caffeic acid (phenylpropanoid moiety) and 4,5-hydroxyphenylethanol (phenylethanoid moiety) bound to a b-(d)-glucopyranoside [98,99]. Recently, it has been demonstrated that it promotes skin repair and ameliorates skin inflammation due to its ROS-scavenging, antioxidant, iron-chelating and glutatione-*S*-transferase (GST)-inducing properties [100]. A rat model of periodontitis showed interesting effects in relation to periodontal tissues for this compound. In this study, oral administration of verbascoside (in this case, biotechnologically produced by *Syringa vulgaris* plant cell cultures) exerted an anti-inflammatory role and ameliorated the tissue damage associated with ligatures placement. The reduction of damage was related to a reduction in oxidative stress levels, which could be mediated by the observed reduction in myeloperoxidase (MPO), iNOS and NO levels. It seems that both effects may be due to verbascoside preventing the ligature-induced IkB-α (nuclear factor of kappa light polypeptide gene enhancer in B-cells inhibitor, α) degradation and consequently also nuclear factor-κB (NF-κB) translocation to the nucleus in gingivomucosal tissue (To have a representation of main cell signaling routes described along this text, see Figure 1) [98]. Verbascoside is also found in olive fruits [101], but because of it is a water-soluble compound, its presence in virgin olive oil probably is much reduced compared with other phenolic compound as hydroxytyrosol.

## 9. Flavonoids

Flavonoids constitute a group of naturally occurring polyphenolic compounds found in a large number of plants and vegetables [102] that have structures consisting of three rings (two of them aromatic) which are labeled A, B and C [103]. More than 4000 varieties of flavonoids have been identified, many of which are responsible for the attractive colors of flowers, fruits and leaves [102]. These can be divided into five subcategories: flavonols, flavones, flavanols, flavanones and anthocyanidines [26,104,105]. Different flavonoid classes differ in the levels of oxidation and pattern of substitution of the C ring, whereas individual compounds within a class differ in the pattern of substitution of the A and B rings [103]. They possess a wide spectrum of biologic activities, including antioxidant, anticarcinogenic, antiangiogenic, anti-inflammatory, antiallergic and antiviral properties [103,106,107,108]. Because of these, different flavonoids have been studied in relation to periodontal diseases as therapeutical or preventive agents. The major compounds researched in this topic and belonging to the different flavonoid subcategories are presented below.

### 9.1. Flavonols

#### 9.1.1. Quercetin

Quercetin (3,5,7,3ʹ,4ʹ-pentahydroxyflavone) is an abundant flavonol-type flavonoid [109,110]. Foods rich in quercetin include apples, black and green tea, onions, red wine, red grapes, citrus fruit, broccoli and other leafy green vegetables, cherries, and a number of berries including raspberry, lingonberry and cranberry [111]. The effects of quercetin on a variety of inflammatory processes and immune functions have been reviewed [112,113,114]. Moreover, the United States Food and Drug Administration (FDA) has approved it as an ingredient in over-the-counter health supplements. The generally recommended adult dose of quercetin for general health benefits is 200–500 mg daily, and up to 1000–1500 mg a day can be consumed [112] and to date, there has been no evidence in the literature of any adverse effects from consuming quercetin [110]. Interestingly, it has been observed that quercetin has potent local osteogenic effects in rabbits [115]. At the periodontal level, it has been demonstrated that the oral administration of Quecetin inhibits bone loss, as well as inflammation, in rats with periodontitis induced by ligatures [116]. Protective effect of quercetins on alveolar bone loss was also reported in a mouse periodontitis model induced by *A. actinomycetemcomitans* infection [109]. This effect has also been related to lower active osteoclasts induced by LPS [116]. This effect seemed due to modulation of cytokine production at least in part, since it was accompanied by a decrease of interleukin IL-1β, tumor necrosis factor-α (TNF-α), IL-17, receptor activator of NF-κB ligand (RANKL), and inducible cell adhesion molecule (ICAM-1) in the gingival tissue, but it has no effect on bacterial counts [109]. Quercetin also has shown a clear antioxidant effect in the oral cavity against NO, which results of nitrite reduction by certain oral bacteria. This occurs at expense of quercetin oxidation, so its effect disappears when all quercetin is oxidized. For this reason, it has been suggested that quercetin can protect human oral cavity from damage induced by RNS and that the protective function of quercetin may be significant when antioxidant capacity of saliva is decreased by periodontal diseases [117]. Consequently, quercetin is an antioxidant molecule with a potential anti-inflammatory action [94,118,119] also on periodontal tissue. For this reason it has been suggested that it may have an ameliorative effect on periodontal destruction. [116].

#### 9.1.2. Kaempferol

Another flavonol, kaempferol (3,5,7-trihydroxy-2-[4-hydroxyphenyl]-4H-1-benzopyran-4-one), which is widely found in fruits, vegetables and tea [120] has also shown strong antioxidant and anti-inflammatory properties [107,108,120] among a variety of pharmacologic activities [120]. Specifically, there is increasing evidence that kaempferol is potentially immunomodulatory in LPS-stimulated cells. Kaempferol was able to reduce cytokines and chemokines produced by LPS-stimulated dendritic cells [121] and inflammatory mediators induced by LPS in microglial cells as well [122]. This suggests that kaempferol has a potential in the treatment of chronic inflammatory and autoimmune diseases [121,122]. Likewise, it inhibited LPS-induced production of monocyte chemoattractant protein-1 (MCP-1), which is involved in the pathogenesis of cardiovascular and neurodegenerative disorders, in macrophages [123]. However, the potential of kaempferol as a therapeutic agent for treating periodontal disease is little studied. In that sense, it has been reported that kaempferol strongly inhibits LPS-induced production of NO at the translational level by *Prevotella intermedia*, another periodontopathogen [124]. In an *in vitro* study with *P. intermedia* LPS-stimulated RAW246.7 cells, this compound significantly inhibited NO production via HO-1-mediated intracellular ROS reduction since it had not affect iNOS mRNA expression, but upregulated HO-1. In fact, inhibition of HO-1 activity by tin protoporphyrin IX (SnPP) abolished the suppressive effect of kaempferol on the production of NO and intracellular ROS [124].

#### 9.1.3. Isorhamnetin

Isorhamnetin (3ʹ-methoxy-3,4ʹ,5,7-tetrahydroxyflavone) is a flavonoid found in apples, blackberries, pears and sea buckthorn [125] and it is also an immediate metabolite of quercetin, at least in mammals [126]. Isorhamnetin has been reported to have many biological properties, including antioxidant and anti-inflammatory activities [127,128]. As consequence it has been tested as potential therapeutic agent in periodontal disease. In RAW264.7 cells stimulated with *P. intermedia* LPS, isorhamnetin significantly down-regulated *P. intermedia* LPS-induced production of IL-6 as well as its mRNA expression, which suggests that this compound may contribute to blockade of some host-destructive processes, at least of those mediated by IL-6. As with the previous flavonols, an up-regulation of HO-1 expression was also noted, which occurred at both transcriptional and translational levels. This seems one of the pathways of the inflammatory effect, since inhibition of HO-1 activity by tin protoporphyrin IX blocked the inhibitory effect of isorhamnetin on IL-6 production. However, isorhamnetin failed to prevent LPS-induced activation of either c-Jun *N*-terminal kinase (JNK) or p38 pathways. On the other hand, it suppressed NF-kB signaling through inhibition of nuclear translocation and DNA binding activity of NF-kB p50 subunit by attenuation of signal transducer and activator of transcription 1 (STAT1) signaling [129].

### 9.2. Flavones

#### 9.2.1. Luteolin

Luteolin (3,4ʹ,5,7-tetrahydroxyflavone) is a flavone found at high concentrations in celery, green pepper, parsley, perilla leaf and seeds and chamomile. As for other flavonoids, a range of biologic and pharmacologic activities of luteolin have been reported [130]. Actually, it has been described as the most potent and efficient inhibitor of TNF-α, IL-6, and NO expression in LPS-stimulated macrophages [131]. For this reason, its efficacy as potential anti-inflammatory compound to treat periodontal disease has been also studied *in vitro*. In human gingival fibroblasts [132] and in murine RAW264.7 cells [26], luteolin pretreatment suppressed LPS-induced NO synthesis, which had been reported to occur in a dose-dependent fashion [26]. Moreover, its effects also include an inhibition of IL-6 release in the macrophage-like cells [132]. These effects were related to a reduction of various mitogen-activated protein kinase (MAPK) family members (including extracellular signal-regulated kinase [ERK], p38, and Akt) activation by phosphorylation [132], which finally prevents NF-κB translocation to the nucleus [26,132]. Furthermore, a reduction of NF-κB p50 subunit DNA binding activity and a suppression of the signal transducer and activator of transcription (STAT) pathway signaling [26] have also been observed. Consequently, iNOS expression would be inhibited explaining the effects of luteolin on NO levels. In addition, luteolin treatment blocked LPS-induced cellular proliferation inhibition without affecting genetic material integrity in fibroblasts [132], but it remains unknown if this was due to its actions on a different pathway.

#### 9.2.2. Baicalin

Baicalin (7-glucuronic acid, 5,6-dihydroxy-flavone) is a flavonoid compound purified from the medicinal plant, *Scutellaria baicalensis georgi* [133,134]. Baicalin has also been found to exert several biological activities, which are mainly related to its antioxidant properties and to its ability to inhibit enzymes and regulate the immune response [133,135,136,137,138]. In addition, it has been used as an anti-inflammatory agent in the treatment of a variety of inflammatory diseases [135,139], which could extend to peridontal diseases as suggested in an experiment in animals. In this, rats with periodontitis induced by ligatures, presented less alveolar bone loss and a higher area fraction of collagen fibers when they were fed baicalin by oral gavage, which correlated with down-regulation of protein levels for cyclooxygenase-2 (COX-2) and iNOS [135]. Similarly, baicalin has been found to inhibit COX-2 activity in a castor-oil-induced diarrhea model, as well as to inhibit iNOS expression in LPS-treated RAW 264.7 cells [140,141]. 

#### 9.2.3. Apigenin

Apigenin, has been found to possess various anti-inflammatory activities useful for different pathologies [26,142,143]. Specifically, apigenin inhibits NO and iNOS synthesis a well as production of several proinflammatory mediators (such as prostaglandin E2, COX-2, and TNF-α), through the suppression of the NF-κB pathway or the STAT pathways [144,145,146,147,148]. In nicotine- and LPS-stimulated human periodontal ligament cells, incubation with apigenin significantly inhibited the nicotine- and LPS-induced production of NO and the up-regulation of iNOS. Moreover, as with other compounds, it was also accompanied by an inhibition of the COX-2 and several proinflammatory factors (PGE2, IL-1β, TNF-α, IL-6, and IL-12) up-regulation. These effects have been attributed to a decrease of LPS- and nicotine-induced HO-1 protein expression and activity, since the selective HO-1 inducer Hemin reversed the apinengin effect on NO, PGE2 and cytokine production were reversed. On the other hand, inhibitors of the phosphoinositide 3-kinase, p38, JNK, and protein kinase C also blocked the anti-inflammatory effects of apigenin in these cells [144]. In spite of the understanding of this control pathway, data regarding its effect on periodontal tissue inflammation or breakdown are not available.

#### 9.2.4. Nobiletin and Tangeretin

Nobiletin (5,6,7,8,3′,4′-hexamethoxy flavone) and tangeretin (5,6,7,8,4′-pentamethoxy flavone) are abundantly present in orange peel, and they exhibit several biological activities [149,150]. In particular, nobiletin has shown to be useful for the treatment of pathologies related to inflammatory disorders [149,151]. Nobiletin markedly suppresses bone resorption and restores the bone loss in ovariectomized mice, a model of postmenopausal osteoporosis [152]. In a short communication, it has been reported that both compounds suppress LPS-induced osteoclast formation and bone resorption and suppress the RANKL-induced osteoclastogenesis in RAW264.7 cells. Moreover, nobiletin clearly restored the alveolar bone mass in a mouse periodontitis model for periodontitis by inhibiting LPS-induced bone resorption. These results indicate that the use of both flavonoids constitutes a new therapeutic approach for periodontal bone loss [150], but more details about their effect are needed.

### 9.3. Flavans

#### 9.3.1. Catechins

Catechins are naturally occurring polyphenolic compounds that are very numerous in green tea to which beneficial effects on human health have been largely attributed [153,154,155]. Research to date suggests that catechins have various biological effects including cardiovascular disease prevention [156] and increase of glucose tolerance [157]. Among others, these compounds have shown antibacterial [158], anti-inflammatory, antioxidant [159] and free radical scavenging properties [160,161], which are interesting for periodontal diseases management. It has even been reported that they possess potent antioxidant activity several times higher than that of vitamin C and vitamin E [162]. Among the most representative catechins are: (−)-epigallocatechin-3-gallate (EGCG), (−)-epigallocatechin(−)-epicatechin-3-gallate, (−)-epicatechin and (+)-catechin. EGCG is the most abundant and biologically active [163]. Specifically, EGCG has many pharmacological effects that have been reported to promote overall health. Actually, the antioxidant and free radical scavenger properties of EGCG are believed to be primarily responsible for the protective effect of tea consumption against the risks of cancer, neuron degeneration and coronary artery diseases [164,165,166]. EGCG alone has been reported to exert a variety of biological effects, including anti-oxidant, antibacterial, anti-inflammatory and anticarcinogenic activities [167,168,169,170].

As for its anticancer effects, EGCG is known to induce apoptosis in various types of tumor cells, but has little or no effect on normal cells [171,172,173]. More recently, it was reported that EGCG also could induce the apoptotic cell death of osteoclast-like multinucleated cells in a dose dependent manner [174]. EGCG has been shown to modulate caspase activation [175,176], but the precise mechanism by which EGCG induces apoptosis and modulates caspase activation remains to be elucidated. In addition, several *in vitro* assays have also reported that EGCG inhibits osteoclast formation [93,177,178]. Regarding possible mechanisms under this effect, Oka *et al.* [177] using osteoclast precursor cell found that EGCG had an inhibitory effect on formation and differentiation to osteoclasts which occurred via matrix metalloproteinase (MMP) suppression. Likewise, Kamon *et al.* [178] reported that EGCG reduced osteoclast formation by inhibiting osteoblast differentiation, since osteoblasts are known to be involved in the differentiation of osteoclasts through the production of RANKL. Such effects of catechins would be useful for prophylaxis or treatment of inflammatory bone disease, such as periodontitis [179]. Actually, catechins, mainly EGCG, have contributed to the prevention [180] and healing [181] of periodontal tissue destruction in different animal models in spite of different administration methods having been tested, including oral administration [180,182], gingival [182] or intraperitoneal injections [181] and topical application as a component of a dentrifice [180]. In most of cases, a decrease of osteoclast number and activity was also observed [181,182,183], which could be due to the effects of catechins on the pathways mentioned. Additionally, it has been noted that these positive effects were also related to decreased gingival oxidative stress, at least at the lipidic and proteic levels [180]. Further, some evidence in humans also supports this interest in catechins. An epidemiological study indicated an inverse association between green tea intake and clinical periodontal parameters (probing depth, clinical attachment level and bleeding on probing) [184], which could be due, as other effects, to its catechins content. In the same sense, a clinical study also reported that the slow-release local delivery of green tea catechins strengthened the effects of traditional periodontal treatment on improvement of periodontitis [185]. Along with the effect on osteoclasts, catechins also have shown to inhibit some enzymatic activities related to periodontal tissue breakdown. In skin, EGCG has been shown to inhibit the infiltration of leukocytes and myeloperoxidase activity along with a decrease UV-B induced erythema [155]. EGCG has been shown to inhibit the activity [186,187,188,189] and expression [190,191] of collagenase (MMP-2) and gelatinase (MMP-9). 

Catechins also show an important role in suppression of inflammatory infiltrate in periodontal tissue after periodontal disease induction [181,183]. Such an effect on inflammation has also been observed in other tissues [155,192]. This anti-inflammatory effect has been related to reduction of pro-inflammatory cytokine levels, namely of IL-1β [182,183], IL-6 [172,193,194] and TNFα [180,183,195], which seems controlled at the transcriptional level [183,195,196]. Similarly, inhibition of the production of these pro-inflammatory cytokines in response to bacterial factors such as LPS has been reported in different *in vitro* assays [197]. Moreover, it has been reported that catechins enhance the production of the anti-inflammatory cytokine IL-10 [195,196]. Down-regulation of the expression of pro-inflammatory cyokines such as TNFα, IL-1β and IL-6 would lead to a decrease in osteoclast number and activity, which results in reduced bone loss [183]. Moreover, in a murine model a decrease of Cyr61-synthesizing osteoblasts has been reported, which subsequently would diminish macrophage chemotaxis into the lesions [181]. The effect of EGCG on Cyr61 production deserves further investigation because Cyr61 might be involved in inflammatory bone loss by promoting pathogenic angiogenesis and macrophage recruitment [181]. In fact, EGCG has been demonstrated to inhibit angiogenesis by suppressing the activation of vascular endothelial growth factor (VEGF) receptor in endothelial cells [142,198].

#### 9.3.2. Theaflavins

Theaflavins are the major polyphenols in black tea. These compound are categorized into the following forms: theaflavin, theaflavin-3-gallate, theaflavin-3ʹ-gallate, and theaflavin-3,3ʹ-digallate (TFDG). In particular, it has been reported that TFDG has stronger biological effects, including an antioxidant effect, compared with other theaflavins [193,199]. As previous compounds, TFDG prevented TNF superfamily 14-mediated IL-6 production in human gingival fibroblast cultures by similar inhibitory actions on ERK, JNK and NF-kB activation as well as receptor expression of the tumor necrosis factor superfamily 14 [200]. Thus, it could also suppress NO production by the iNOS expression inhibition. This suggests that theaflavins, namely TFDG, could be used as catechins for periodontal disease management, but there is need for more studies with these compounds.

#### 9.3.3. Proanthocyanidins

Proanthocyanidins are among the most abundant phenolic compounds in grape seeds (*Vitis vinifera*). These molecules have been associated, at least in part, with the protective effect of red wine for atherogenesis and cardiovascular diseases [201,202]. In support of this, recent studies have shown that oligomeric proanthocyanidins in grape seeds prevent heart disease and skin aging [203], and that they possess anti-inflammatory, antiarthritic and antitumor-promoting activities [204]. As other compounds reviewed, possible mechanisms by which these phenolic compounds might exert their protective effects include antioxidant properties, oxygen and nitrogen scavenging abilities [201,202], and inhibitory effects on both arachidonic acid cascade [201] and prostaglandins modulation [204,205]. Interestingly, grape seed proanthocyanidins have been shown to exert a much stronger oxygen-free radical scavenging effect than vitamins C and E [206]. Several *in vitro* assays have offered similar result in relation to periodontal disease. In murine RAW 264.7 cells, treatment with non-toxic concentrations of grape seed proanthocyanidin extract, strongly decreased NO and ROS production along with iNOS protein expression after simulation with LPS of two major periodontopathogens, *A. actinomycetemcomitans* or *F. nucleatum* [60]. Likewise, it was found that a proanthocyanidin-rich cranberry fraction possessed the capacity to reduce the production of inflammatory mediators by LPS-stimulated macrophages and gingival fibroblasts [207,208] and to inhibit host extracellular matrix destructive enzyme production and activity [209]. In addition, the same cranberry fraction could prevent biofilm formation by *Porphyromonas gingivalis* [210] a major etiological agent of chronic periodontitis. Given these results, proanthocyanidins should be considered a potential agent in the prevention of periodontal disease.

## 10. Conclusions

The present review was performed to explore whether naturally occurring non-nutrient phenolic compounds present in various foods and plants could be useful for periodontal disease because of their antioxidant effect or activity. In this context, *in vitro* studies have shown that many phenolic compounds in foods and their derivatives could help to reduce oxidative stress triggered by the presence of periodontal bacterial products (in particular by LPS). This seems largely due to immunomodulatory actions [130,131,208,210], to the promotion of the antioxidant enzymes activity, such as HO-1 [95,125,130,145], and/or to the inhibition of enzymes that contribute to RNS and ROS production such as iNOS [61,125,142,194,210]. Although the number of compounds used in animal models has been low, these studies have allowed to observe that many of the compounds improve or prevent the development of this pathology both in terms of periodontal inflammation [26,51,67,83,117,181] and periodontal tissue breakdown [26,29,51,83,93,110,136,183,184]. In turn, these effects have been shown to be associated with lower levels of nitro-oxidative stress [93,130,181] and proinflammatory mediators [83,93,110,181,182,183,184]; and consequently with lower levels of oxidative damage [18,93]. However, very few studies evaluating their actions in humans are reduced only to CoQ [39,47,52] and catechins [186] that in most of cases were combined with previous mechanical treatments [39,52,186]. Apart from these, the sample sizes used were small. Hence, many more interventions in humans are needed to evaluate the possible effects of different compounds on periodontal diseases, particularly those that are more ubiquitous, although for ethical reasons, these compounds should be combined with other treatments. In this sense, epidemiological studies evaluating the content in the diet of these compounds could also be very interesting to identify possible targets before performing experimental trials.

**Table 1:** Main studies in relation to naturally occurring antioxidant phenolic compounds of interest in periodontal disease.

Compound	Study Type	Sample Studied (age/weight), *n*	Adminitration (Dosage, Frecuency and Duration)	Main Effect/s	Reference
Thymoquinone	Animal	Male Wistar rats with ligature-induced periodontitis (300 ± 10 g), *n* = 8	Intragastric (10 mg/kg, daily for 11 days)	ABL, inflammatory infiltrate and osteoclasts *n*° were reduced; osteoblasts activity was maintained	[25]
Coenzyme Q	NCT	Patients under routine care for periodontitis (N/A) *n* = 8	Oral (N/A)	Periodontal inflammation and periodontal pocket depth decreased	[46]
	RCT	Chronic periodontitis patients (20–55 year), *n* = 3–6	Topical (CoQ_10_ in a vegetable oil base in ratio of 1:9 in gel-form for 2/4 weeks)	Plaque index, gingival index, gingival bleeding index, periodontal probing pocket depth, and clinical attachment level were improved	[38,51]
	Animal	Male Fischer rats without additional treatments (8/16 weeks) *n* = 24	Topical (ointment with 1% rCoQ10 daily for 2/4 months)	Osteoclast differentiation associated to aging decreased. The same conditions lowered gene periodontal expression of caspase-1 and IL-1β	[50]
Gallic acid	*In vitro*	RAW 264.7 cells stimulated with LPS of *A. actinomycetemcomitans* or *F. nucleatum*	Preincubation (4 mg/ml for 2 h)	NO production was strongly inhibited	[60]
Hidroxytyrosol	Animal	New Zealand White rabbits with dietary-induced atherosclerosis (2.5 kg), *n* = 8	Dietary supplementation (25 mg/kg daily for 30 days)	Endothelial activation was decreased	[66]
Mangiferin	Animal	Male Wistar rats with ligature-induced periodontitis (180 g), *n* = 4–7	Oral (100 mg/kg daily for 7 days)	ABL and cellularity were reduced. COX-2 expression and the inhibition of rolling and adhesion of leukocyte	[82]
Resveratrol	Animal	Male Wistar rats with ligature-induced periodontitis (8 weeks), *n* = 6	Oral supplementation (10 mg/kg daily melinjo resveratrol in drinking water for 20 days)	ABL was prevented and systemic levels of 8OH-dG, dityrosine, nitrotyrosine and proinflammatory cytokines (IL-1β IL-6, and TNF-α) were reduced	[92]
	Animal	Male Wistar ratswith ligature-induced periodontitis (12 weeks), *n* = 12	Intragastric (10 mg/kg daily for 30 days)	ABL and gingival IL-17 were reduced, but L-1β and IL-4 levels were unaffected	[28]
Verbascoside	Animal	Male Sprague–Dawley rats with ligature-induced periodontitis (280–400 g), *n* = 10	Oral (2 mg/kg daily for 8 days)	Gingivomucosal tissue injury and multiple inflammation and oxidative stress markers (MPO activity, activation of poly(ADP-ribose) polymerase TBARS, nitration of tyrosine residues, expression of NF-κB , iNOS, Bax and Bcl-2) were decreased	[98]
Quercetin	Animal	Male Sprague–Dawley rats LPS-injected (6 weeks), *n* = 4	Oral (75 mg/kg, daily for 8/12 days)	Osteoclasts induction and inflammatory infiltrate were reduced. Bone crest levels were more apical and coronal alveolar level was higher than in the controls	[116]
	Animal	Male Balb/c mice with *A. actinomycetemcomitans* infection (6–7 weeks), *n* = 5	Subcutaneous injections (100 mg/kg daily for 15 days)	ABL and gingival levels of IL-1β, TNF-α, IL-17, RANKL, and ICAM-1 were reduced	[109]
Kaempferol	*In vitro*	RAW246.7 cell stimulated by LPS from *P. intermedia*	Coincubation (10–100 μM for 24 h)	NO production was strongly inhibited	[124]
Isorhamnetin	*In vitro*	RAW264.7 cells stimulated by LPS from *P. intermedia*	Coincubation (12.5–50 μM for 24 h)	IL-6 mRNA and protein levels were down-regulated	[129]
Luteolin	*In vitro*	RAW264.7 cells stimulated by LPS from *E. coli*	Preincubation (10 μM for 30 min/60 min)	Activation of several MAPK family members and TNF-a release were reduced	[131]
	*In vitro*	Human gingival fibroblasts stimulated by LPS from *Salmonella enteritidis*	Preincubation (10 μM for 30 min)	NO synthesis and cellular proliferation inhibition were suppressed	[132]
Baicalin	Animal	Male Sprague–Dawley rats with ligature-induced periodontitis (8 weeks), *n* = 9 groups	Intragastric (50, 100 or 200 mg/kg, daily for 7 days)	ABL and collagen fibers loss were reduced; which was associated with COX-2 and iNOS levels down-regulation	[134]
	*In vitro*	Human periodontal ligament cells and gingival fibroblats stimulated by IL-1β	Coincubation (N/A for 1 h)	Pro-MMP-1 secretion and MMP-3 expression were reduced	[136]
	*In vitro*	RAW 264.7 cells stimulated by LPS	Coincubation (5–40 μM for 24 h)	iNOS expression was reduced	[141]
Apigenin	*In vitro*	Human periodontal ligament cells stimulated by nicotine and LPS from *P. gingivalis*	Preincubation (40 μM for 4 h)	NO production and up-regulation of iNOS, COX-2 and several proinflammatory factors (PGE2, IL-1β, TNF-α, IL-6, and IL-12) were inhibited	[144]
Nobiletin and tangeretin	*In vitro*	RAW264.7 cells stimulated by LPS	(30 μM for 5 days) in the presence of sRANKL (100 ng/mL)	Osteoclast formation and bone resorption and RANKL-induced osteoclastogenesis was reduced	[150]
Catechins	RCT	Volunteers with advanced periodontitis but with no systemic disorders (41–64 years), *n* = 6	Topical (HPC strips containing GTC, once a w for 8 weeks)	Pocket depth and the proportion of BPR were decreased in combination with mechanical treatment	[185]
	Animal	Male Sprague–Dawley Rats with ligature-induced periodontitis (150–180 g), *n* = 24	Intragastric (200 mg/kg EGCG daily for 1/2/4 weeks)	ABL and collagen destruction were decreased which was associated to a reduction of IL-6 and TNF-α expression	[183]
	Animal	Male Wistar rats with periodontal inflammation induced by LPS from *E. coli* and proteases from *S. griseus* (8 weeks), *n* = 11	Topical (dentifrice with 1.0% GTC daily for 4 weeks)	Inflammatory infiltrate was reduced which was accompanied by lower gingival hexanoyl-lysine, nitrotyrosine, and TNF-α	[180]
	Animal	Male BALB/c mice receiving gingival injections of LPS from *E. coli* (7 weeks), *n* = 6	Oral supplementation (in drinking water with 1% Sunphenon BG ^1^ free acess for 20 days)	ABL and IL-1β expression were decreased	[182]
	Animal	Male BALB/c mice receiving gingival injections of LPS from *E. coli* (7 weeks), *n* = 6	Gingival injection (1% Sunphenon BG ^1^ every 48 h for 20 days)	ABL and IL-1β expression were decreased	[182]
	*In vitro*	Osteoclasts from mouse bone marrow macrophagesstimulated by RANKL	Coincubation (2–10 μg/mL Sunphenon BG ^1^ for 4 days)	Osteoclasts *n*° decreased in a dose-dependent manner	[182]
	*In vitro*	RAW264.7 cells stimulated by LPS from *E. coli*	Preincubation (50 mmol/L of EGCG for 2 h)	TNFα production and mRNA expression were decreased as well as nuclear NF-κB–binding activity	[195]
	*In vitro*	Human gingival fibroblasts stimulated by oncostatin M	Preincubation (5/50 μg/mL EGCG or ECG, for 1 h)	CXCL10 production was prevented	[193]
	*In vitro*	Human gingival fibroblast stimulated by tumor necrosis factor superfamily 14	Preincubation (5/50 mg/mL EGCG or ECG for 1 h)	IL-6 production was prevented	[199]
Theaflavins	*In vitro*	Human gingival fibroblast stimulated by tumor necrosis factor superfamily 14	Preincubation (5/50 mg/mL TFDG for 1 h)	IL-6 production was prevented	[199]
Proanthocyanidins	*In vitro*	Human macrophages stimulated by LPS from different periodontopathogens ^2^	Preincubation (10–50 μg/mL of a Proanthocyanidin-rich cranberry fraction for 2 h)	IL-1β, IL-6, IL-8, TNF *-*α, and RANTES production was reduced	[206]
	*In vitro*	Human gingival fibroblasts stimulated by LPS from *A. actinomycetemcomitans*	Preincubation (10-50 μg/mL of a Proanthocyanidin-rich cranberry fraction for 2 h)	IL-6, IL-8, and PGE2 production and increase of COX-2 expression were inhibited	[207]
	*In vitro*	Human macrophages and gingival fibroblasts stimulated by LPS from *A. actinomycetemcomitans*	Preicubation (10–150 μg/mL of a proanthocyanidin-rich cranberry fraction for 2 h)	MMP-3 and MMP-9 production and activity were inhibited	[208]
	*In vitro*	RAW 264.7 cells, simulated by LPS from *A. actinomycetemcomitans* or *F. nucleatum*	Grape seed proanthocyanidin extract (4 mg/mL for 2 h)	NO and ROS production and iNOS l levels decreased	[60]

^1^ Sunphenon BG contains 91.3% polyphenol and 76.6% catechins, consisting of 45.9% EGCG, 9.6% epigallocatechin, 8.6% ECG, 5.3% epicatechin and others; ^2^ *A. actinomycetem comitans*, *F. nucleatum* subsp. *Nucleatum*, *P. gingivalis*, *Treponema denticola*, *Tannerella forsythia*, and *E. coli.* Abbreviations and acronyms: 8OH-dG: 8-hydroxydeoxiguanoxine; *A. actinomycetemcomitans*: *Aggregatibaceter actinomycetemcomitans*; ABL: Alveolar bone loss; BPR: Gram-negative anaerobic rods; COX-2: Cyclooxygenase-2; CXCL10: C-X-C motif chemokine 10; *E. coli*: *Escherichia coli*; ECG: Epicatechin gallate; EGCG: Epigallocatechin gallate; *F. nucleatum: Fusobacterium nucleatum*; GTC: Green tea catechins; h: hours; HPC: Hydroxypropyl cellulose; ICAM-1: Intercellular adhesion molecule; IL: Interleukin; iNOS: Inducible nitric oxide synthase; LPS: Lypopolysaccharide; MMP: Matrix metalloproteinase; MPO; Myeloperoxidase; *n*: sample size per group; N/A; not available; NF-κB; Nuclear factor κB; NTC: non-controlled trial; *P. gingivalis*: *Porphyromonas gingivalis*; *P. intermedia*: *Prevotella intermedia*; PGE2: Protaglandin E2; RANKL: Receptor activator for nuclear factor κ B ligand; RANTES: Regulated on activation normal T-cell expressed and secreted; ROS: Reactive oxygen species; RCT: Randomized controlled trial; *S. griseus*: *Streptomyces griseus*; TBARS: Tiobarbituric acid reactive sustances; TFDG: Theaflavin digallate; TNF-α: Tumor necrosis factor-α.
